# Reirradiation of locally recurrent prostate Cancer: a review of prospective evidence and delineation and planning strategies from the reirradiation Collaborative group (ReCOG)

**DOI:** 10.1016/j.ctro.2026.101229

**Published:** 2026-07-15

**Authors:** Finbar Slevin, Brad Warkentin, Jeremy Hansen, Ann M. Henry, Louise J. Murray, Donna H. Murrell, Wee Loon Ong, David Pasquier

**Affiliations:** a Leeds Institute of Medical Research, University of Leeds, UK and Leeds Teaching Hospitals NHS Trust, Leeds, UK; b Department of Oncology, Division of Medical Physics, University of Alberta; Department of Medical Physics, Cross Cancer Institute, Cancer Care Alberta; Edmonton, Alberta, Canada; c Varian Medical Systems, Housten, TX, USA; d Department of Oncology, Western University, London, Ontario, Canada; Verspeeten Family Cancer Centre, London Health Sciences Centre, London, Ontario, Canada; e Alfred Radiation Oncology, Bayside Health, School of Translational Medicine, Monash University, Melbourne, Victoria Australia; Department of Radiation Oncology, Andrew Love Cancer Centre, Barwon Health, Geelong, Victoria, Australia; School of Public Health and Preventive Medicine, Monash University, Melbourne, Australia; f Academic Department of Radiation Oncology, Centre Oscar Lambret, Lille, France; Lille University, CRIStAL UMR CNRS 9189, Lille, France

**Keywords:** Prostate cancer, local recurrence, radiorecurrent, reirradiation, re-irradiation, stereotactic body radiotherapy, stereotactic ablative radiotherapy, brachytherapy

## Abstract

There is considerable uncertainty regarding the optimum approaches to target volume and organ at risk (OAR) delineation and tolerances to reirradiation for locally recurrent prostate cancer. To address these uncertainties, we undertook a literature review of treatment planning approaches of stereotactic body radiotherapy (SBRT) or brachytherapy reirradiation in published and ongoing prospective studies. Twenty published and 11 ongoing studies were identified. No phase 3 clinical trials were identified. Both focal and whole gland with/without simultaneous integrated boost approaches to target volume delineation were used. SBRT dose-fractionation schedules ranged from 25 to 42.5Gy in 5 fractions and 36-38Gy in 6 fractions with varying approaches to target objectives. Brachytherapy studies utilised both low dose rate and high dose rate approaches and employed a range of dose-fractionation schedules. Approaches to OAR delineation and constraints were heterogenous. No published and only 1 ongoing study utilises cumulative OAR constraints. Acceptable rates of genitourinary and gastrointestinal toxicities were observed in the majority of studies although some studies reported relatively high rates of severe toxicity events. The relatively short follow-up of some published series may underestimate the incidence of late toxicities. There remains uncertainty regarding optimum dose-fractionation schedules, target volumes and OAR constraints and how these influence toxicity. Prostate reirradiation using SBRT or brachytherapy can be used for treatment of locally recurrent prostate cancer. Treatment is recommended within prospective studies or registries with robust target definition, treatment delivery and comprehensive assessment of toxicity so that evidence to optimise treatment can be generated.

## Highlights

• Review of prospective studies of reirradiation for prostate cancer local recurrence.
• Stereotactic body radiotherapy and brachytherapy techniques utilised.
• Heterogenous approaches to target delineation and objectives.
• Heterogenous approaches to organ at risk delineation and constraints.
• Acceptable rates of urinary and bowel toxicities observed in most studies.

## 1 Introduction

Prostate cancer is the most commonly diagnosed cancer in men across Europe and North America. Most patients present with localised/locally advanced disease which can be treated with curative intent using surgery with/without post-operative radiotherapy or definitive radiotherapy and/or brachytherapy [1].

Despite this, biochemical recurrence, demonstrated by a rising prostate specific antigen (PSA) post radical therapy, remains common. With increasing use of positron emission tomography-computed tomography (PET-CT) during early biochemical recurrence, patients with isolated local disease recurrence can be identified [2]. An estimated 3–13% of patients may experience isolated local recurrence following definitive radiotherapy [3], [4], [5], [6], [7].

There is potential that salvage therapies, such as prostatectomy, cryotherapy, high frequency ultrasound (HIFU) or reirradiation using brachytherapy or stereotactic body radiotherapy (SBRT), could offer a second chance of cure or provide long-term disease control for patients with locally recurrent prostate cancer following prior definitive radiation. Observational data suggest disease-related outcomes are similar between these salvage treatments but that reirradiation with brachytherapy or SBRT may result in lower risk of severe toxicity, especially genitourinary (GU) toxicity [5].

With the increasing interest in salvage reirradiation for locally recurrent prostate cancer, clinical practice guidelines are now emerging [8]. Nonetheless, Delphi studies performed with international experts have failed to gain consensus for many treatment-related factors [9], [10]. There is considerable variation in published approaches to target volume/organ at risk (OAR) delineation and target volume dose-fractionation schedules, planning objectives and OAR dose constraints.

To address these uncertainties, we undertook a literature review of treatment planning approaches for patients with locally recurrent prostate cancer treated with reirradiation. The review focused on delineation, dose fractionation schedules and planning objectives from reported and ongoing prospective clinical trials.

## 2 Methods

The review was conducted by the Reirradiation Collaborative Group (ReCOG)’s GU Group [11], [12]. Literature searches were performed between 1st September 2025 and 1st April 2026 using ClinicalTrials.gov and PubMed for terms relating to, but not restricted to, reirradiation, salvage, radiotherapy, brachytherapy and recurrent prostate cancer. Further articles were identified by manually searching reference lists of relevant publications. The search was updated on 16th June 2026, and an additional 2 published studies were identified. Multiple existing systematic reviews have addressed disease and toxicity outcomes for reirradiation of locally recurrent prostate cancer [5], [13], [14], [15], [16]. In contrast, this review focuses specifically on treatment planning related factors and how these relate to toxicity outcomes and was restricted to published and ongoing clinical trials/prospective observational studies.

## 3 Results

### 3.1 Published prospective studies

Initial and reirradiation patient, disease and treatment characteristics for published series using SBRT and brachytherapy for reirradiation of locally recurrent prostate cancer are summarised in Table 1.

**Table 1:** Initial and reirradiation patient, disease and treatment characteristics for prospective published studies.

Study	Year	Type	Number of participants	Type of reRT	Median age (range)	Initial RT technique	Initial RT dose	Staging at reRT	ISUP score at reRT	Median PSA at reRT (ng/ml) PSAdt at reRT	Imaging for local recurrence	Biopsy confirmation of local recurrence	Time interval to reRT	Pre-existing toxicity	Urinary function reRT	ADT with reRT	Median follow up (range)
Bergamin [17]	2020	Prospective cohort	25	SBRT	72 (62–83)	EBRT 84% EBRT + BT 8% BT 8%	EBRT dose ≥78Gy 52% <78Gy 48%	≤T2a 100%	–	PSA 4.1 (1.1–16.6)	PET-CT MRI	Yes	Minimum 4 years Median 8.3 years (4.5–13.6)	No G3+ toxicity	–	No	25 months (13–46)
Buchalet (including Michalet 2022) [18]	2025	Prospective registry	108	SBRT	76 (68–93)	EBRT 73% PORT 23% BT 4%	EBRT 76Gy (70–80) PORT 66Gy (64–66)	≤T2 or prostate bed	ISUP 1–3 54% ISUP 4–5 42%	PSA 3 (0.4–30.5) PSAdt 8.9 months (1.1–57)	PET-CT MRI	Not mandated	Minimum 18 months	No G3+ toxicity	–	Yes in 20%	31.9 months (1.5–63)
Cuccia [21]	2022	Prospective cohort	22	SBRT	66 (51–85)	EBRT 45% BT 9% PORT 45%	–	–	–	PSA 1.7 (0.34–8.58)	PET-CT or MRI	Not mandated	Minimum 12 months Median 72 months (12–146)	–	IPSS <10	Yes in 18%	8 months (3−21)
Ekanger [22]	2024	Prospective cohort	38	SBRT	70 (59–84)	EBRT 100%	78Gy (66–81)	T1–2 89% T3 3%	ISUP 1–3 68% ISUP4–5 32%	PSA 4 (2−23) PSAdt <6 months 18% 6–12 months 34% >12 months 47%	MRI PET-CT in 13%	Yes	Minium 2 years Median 83 months (30–158)	No G2+ toxicity		Yes in 97% for 6 months	86 months (11−111)
Fuller [23]	2020	Prospective cohort	50	SBRT	74 (50–89)	EBRT 88% BT 10% PORT 2%	75.6Gy (35–145)	T1–2 96% T3 2%	ISUP 1–3 62% ISUP4–5 36%	PSA 3.97 (0.1–48.2)	MRI	Yes	Minimum 2 years Median 98 months (31–241)	No G2+ toxicity	–	Yes in 14%	44 months (3−110)
Greco [24]	2022	Prospective cohort	30	SBRT	72 (IQR 77–79)	EBRT 57% EBRT + BT 7% BT 37%	EBRT 74Gy (IQR 71.6–74)	T1–2100% (23% had N1 or M1 oligometastases)	ISUP 2–3 17% ISUP 4–5 27% NK 57%	PSA 3.8 (IQR 2.9–4.8)	PET-CT MRI (in some cases)	Not mandated	Minimum 2 years Median 70.2 months (51.3–116)	No G2+ toxicity		Yes in 53% for median 15 months (IQR 6–34)	44 months (IQR 18–60)
Lewin [25]	2021	Prospective registry	30	SBRT	71 (56–83)	EBRT 83% BT 17%	EBRT: 80Gy (74–82) BT: 145Gy	T1–2 53% T3+ 47% (26% had N1 or M1 oligometastases)	ISUP 4–5 27% NK 73%	PSA 3.63 (0.05–77)	PET-CT MRI	Not mandated	Median 9 years (2–20 years	No G3+ toxicity	–	Yes in 87%	28 months (19–83)
Majewski⁎ [36]	2026	Phase 2	21 (out of planned 55)	SBRT	69	EBRT 67% PORT 33%		GTV <14 cc		PSA 1.3 (0.6–2.6) PSAdt 10.6 months (5.2–12.6)	PET-CT MRI	Not mandated	Minimum 2 years	No G2+ or prior G3+ toxicity	IPSS <19	Yes in 48%	14 months (4.5–25)
Pasquier [28]	2023	Phase 1	21	SBRT	76.8 (IQR 72.2–80.8)	EBRT 100%	74Gy (IQR 74–76)	T1–2100%	ISUP 1–3 85% ISUP 4–5 15%	PSA 4.2 (IQR 3.2–6.0) PSAdt >10 months (median 25.4)	PET-CT MRI	Yes	Minimum 2 years Median 8.6 years (IQR 6.3–10.7)	No G2+ toxicity	IPSS ≤12	No	12.3 months (IQR 9–19.5)
Patel [30]	2023	Phase 1	8	SBRT	64 (53–73)	EBRT 100%	76.5Gy (72–79.2)	T1–3100%	ISUP 4–5 88%	PSA 2.92 (2.05–7.92)	PET-CT MRI	Yes	Minimum 1 year Median 9.1 years (6.3–16.4)	No G3+ toxicity	–	No (except in 1 case due to treatment delay)	27 months (6–43)
Patel [29]	2024	Phase 1	9	SBRT	69 (60–77)	BT 89% EBRT + BT 11%	125Gy (27–145)	T1–3100%	ISUP 2–3 44% ISUP 4–5 22%	PSA 3.1 (2.1–19.5)	PET-CT MRI	Yes	Minimum 1 year Median 8.2 years (3.7–11.0)	No G3+ toxicity	Median IPSS 7 (2−20)	Yes in 11%	22 months (IQR 20–43)
																	
Crook (including Crook 2019) [20]	2022	Phase 2	92	LDR BT	70 (55–82)	EBRT 100%	73.8Gy (45–81)	T1–2100%	ISUP 1–3100%	PSA 7.3 (0.4–19.6)	CT ^99^Tc bone scan (for eligibility not recurrence location)	Yes	Minimum 30 months Median 85.4 months (39.3–199.4)	No G2+ toxicity	IPSS <15	Yes in 16% for up to 6 months	80.4 months (3.6–134.4)
Chitmanee [19]	2020	Prospective cohort	50	HDR BT	70 (57–82)	EBRT 44% BT 50% EBRT + BT 2% HIFU 4%	74Gy in 15 of 22 EBRT patients	T1–2 72% T3 28%	ISUP 1–3 54% ISUP 4–5 36%	PSA ≤10 94% >10 6% PSAdt ≤12 months 34% >12 months 66%	MRI PET-CT in some patients	Not mandated	≤5 years 56% >5 years 44%			Yes in 8%	21 months (1–53)
Ménard [26]	2022	Prospective cohort	88	HDR BT	71 (56–85)	EBRT 72% BT 20% EBRT + BT 8%	78Gy (62–115)	T1–2 77% T3–4 23%	ISUP 1–3 65% ISUP 4–5 35%	PSA 4.5 (1.2–24)	PET-CT MRI	Yes	Minimum 18 months Median 7 years (3–17)		IPSS ≤18	Yes in 30% for up to 6 months	35 months (6–134)
Nguyen [27]	2007	Phase 2	25	LDR BT	65 (56–82)	EBRT 52% BT 44% EBRT + BT 4%	EBRT 66–70.2Gy BT 137Gy	–	–	PSA 5.5 (1.4–11.6)	MRI	Yes	Minimum 2 years Median 5.2 years (2.5–12.8)			No	47 months (14–75)
Paulin (update of Murgic 2018 and Corkum 2022) [32]	2025	Phase 2	59	HDR BT	72 (IQR 66–76)	EBRT 32% BT 54% EBRT + BT 14%			ISUP 1–3 81% ISUP 4–5 19%	PSA 4.1 (IQR 3–6.4)	MRI PET-CT in 42%	Yes	Median 5.3 years (IQR 4–7.5)		IPSS <15	No	54 months (11−132)
Rasing (update of van Son 2020, van Son 2020 and Maenhout 2017) [33]	2023	Prospective cohort	175	HDR BT	72 (IQR 68–75)	EBRT 54% BT 46%		T1–2 55% T3–4 45%		PSA 4.4 (IQR 2.5–6.7) PSAdt 16.6 months (IQR 11.6–24.1)	PET-CT MRI		91.5 months (IQR 62–133)		IPSS <15	No	20 months (IQR 12–31)
Ryg [34]	2022	Prospective cohort	33	HDR BT 70% SBRT 30%	70 (63–78)	EBRT 100%	74Gy (70–78)			PSA 4.1 (0.3–7.9) PSAdt >6 months	MRI PET-CT (79%)	Yes	Minimum 2 years Median 73 months (IQR range 52.5)			Yes in 15%	81 months (21–115)
Strnad [37]	2026	Phase 2	109	HDR BT 34% PDR BT 76%	74 (55–83)	EBRT 100%	70.2Gy (45–78)	T1–2 61% T3 9% NK 30%	ISUP 1–3 17% ISUP 4–5 14% NK 69%	4.0	MRI or TRUS PET-CT or CT/bone scan	Yes	Minimum 2 years	No G2+ toxicity		Yes in 24%	64 months
Yamada [35]	2014	Phase 2	42	PDR BT	72 (56–83)	EBRT 100%	81Gy (68.4–86.4)		ISUP 1–3 67% ISUP 4–5 33%	PSA 3.54	MRI (for eligibility not recurrence location)	Yes	Median 78 months		IPSS <15	Yes in 43%	36 months (6–66)

ADT, androgen deprivation therapy; BT, brachytherapy; CT, computed tomography; EBRT, external beam radiotherapy; EQ.D2, equivalent dose in 2Gy fractions; HDR BT, high dose rate brachytherapy; HIFU, high frequency ultrasound; IPSS, International Prostate Symptom Score; IQR, inter-quartile range; ISUP, International Society of Urological Pathologists; LDR BT, low dose rate brachytherapy; MRI, magnetic resonance imaging; PDR BT, pulsed dose rate brachytherapy; PET-CT, positron emission tomography-computed tomography; PORT, post-operative radiotherapy; PSA, prostate specific antigen; PSAdt, prostate specific antigen doubling time; reRT, reirradiation; SBRT, stereotactic body radiotherapy; TRUS, transrectal ultrasound.

⁎ Majewski et al. study ongoing but published preliminary toxicity results from subset of cohort included here.

#### 3.1.1 Summary of published studies

In total twenty studies were included: Ten using SBRT, 9 using low dose rate (LDR) or high dose rate (HDR) brachytherapy and 1 using both SBRT and HDR brachytherapy [17], [18], [19], [20], [21], [22], [23], [24], [25], [26], [27], [28], [29], [30], [31], [32], [33], [34], [35], [36], [37]. The study by Patel et al. reports quality of life outcomes in the studies by Patel et al. and Patel et al. [28], [29], [30]. Studies using SBRT included 3 phase 1 clinical trials and 1 phase 2 trial with the remainder prospective observational studies/registries [17], [18], [21], [22], [23], [24], [25], [28], [29], [30], [34], [36]. Studies using brachytherapy included 5 phase 2 trials [19], [20], [26], [27], [32], [33], [34], [35], [37]. No phase 3 studies were identified.

Number of patients per study ranged from 8 to 175. Median initial external beam radiotherapy dose was ≥74Gy in equivalent dose in 2Gy fractions (EQ.D2) in most studies. The majority of studies staged patients using both PET-CT and magnetic resonance imaging (MRI). Biopsy confirmation of local recurrence was mandated in 6 SBRT and all but 1 brachytherapy study and not mandated in 5 SBRT studies.

Most studies permitted T1–3 and International Society of Urological Pathologists (ISUP) grade 1–5 disease at reirradiation. Median PSA value ranged from 1.3 to 5.5 ng/ml with only 3 studies stipulating a minimum PSA doubling time (ranging from 6 to 12 months).

Minimum time interval between initial radiotherapy and reirradiation ranged from 1 to 4 years, with median reported interval ranging from 5.2 to 9.1 years. Most studies stipulated no grade ≥ 2 or ≥ 3 pre-existing GU or gastrointestinal (GI) toxicity for reirradiation eligibility. There was varying use of androgen deprivation therapy (ADT) with reirradiation.

#### 3.1.2 Technical characteristics

Technical characteristics are summarised in Table 2.

**Table 2:** Reirradiation technical characteristics for prospective published studies.

Study	ReRT technique	ReRT dose-fractionation	Timing	Preparation	Target	GTV definition	Median or mean GTV volume	CTV definition	Median or mean CTV volume	PTV definition	Median or mean PTV volume	Prescription point	Maximum dose	Prioritisation
Bergamin [17]	SBRT	36Gy/6# 72% 38Gy/6# 28%	Alternate days	Full bladder Empty rectum Fiducials Spacer if lesion adjacent to rectum	Focal	PET-CT/MRI defined lesion	2.7 cc (0.8–8.5)	GTV + 3 mm constrained to prostate/urethra	–	CTV + 3 mm	13.3 cc (4.3–32.2)	PTV D95% ≥100% prescribed dose CTV D95% ≥100% GTV D100% ≥100%	GTV D0.1cc ≤130% PTV D0.1cc ≤115%	
Buchalet (including Michalet 2022) [18]	SBRT	30Gy/5# 62% 36Gy/6# 38%	Alternate days	–	Focal	PET-CT/MRI defined lesion	2.3 cc (0.4–22.3)	–	–	GTV + 3 mm	9.4 cc (2.4–61.8)	PTV D95% ≥95% prescribed dose	–	
Cuccia [21]	SBRT	30Gy/5#	Alternate days 86% Daily 14%	Full bladder Empty rectum	Whole gland	–	–	Prostate or PET-CT defined prostate bed lesion	11.65 cc (0.8–30.3)	CTV + 3–5 mm	23.3 cc (4.8–64.2)	PTV D95% ≥95% prescribed dose	PTV V107% ≤2% prescribed dose	
Ekanger [22]	IMRT	35Gy/5#	Alternate days	Fiducials	Focal in 79% Whole gland in 21%	MRI defined lesion	–	GTV + regions with positive biopsies	–	CTV + 5 mm except posteriorly	–	PTV D98% ≥95% & ≤107% prescribed dose		
Fuller [23]	SBRT	34Gy/5#	Daily	Fiducials	Whole gland	–	–	Prostate + extracapsular extension	–	CTV + 0 mm	21.5 cc (10.5–47.7)	PTV D95% ≥100% prescribed dose	Dmax >150% (minimum Dmax for ‘virtual HDR BT’)	
Greco [24]	SBRT	35-40Gy/5#	Daily 87% Alternate days 13%	Empty rectum Urethral catheter Endorectal balloon	Whole gland 35Gy 60% Whole gland 40Gy 7% Whole gland + SIB 40Gy 27% Focal 40Gy 7%	PET-CT/MRI defined lesion for SIB/focal	–	Prostate for whole gland	26.7 cc (IQR 24.0–39.3)	CTV + 2 mm (0 mm adjacent to rectum, urethra PRV, urogenital diaphragm and neurovascular bundle)	–	PTV D50% ≥35Gy	Planning objective: PTV D2% ≤37.5Gy Achieved: D2% 37.5Gy (IQR 37.3–41.6)	OAR constraints prioritised
Lewin [25]	SBRT	Median 32.5Gy (30–40)/5# 100% Additional pelvic nodal RT 25Gy/5# 23%	Alternate days	Full bladder Fiducials Endorectal balloon 73% Spacer 27%	Whole gland 57% Focal 43%	PET-CT/MRI defined lesion for focal	–	Prostate or GTV + 3–5 mm	–	CTV + 0 mm	–	PTV D98% ≥95% prescribed dose	D2% <110% prescribed dose	
Majewski⁎ [36]	SBRT	33.75Gy/5#	Alternate days	Empty rectum Full bladder	Focal	PET-CT/MRI defined lesion	1.8 cc (1.1–4.6)	GTV + 1–3 mm		CTV + 3 mm (1 mm where PTV overlaps with bladder/rectum)				
Pasquier [28]	SBRT	Dose escalation 25Gy/5# 5% 30Gy/5# 38% 36Gy/6# 57%	Alternate days	Empty rectum Full bladder Fiducials	Focal (CTV <50% prostate by volume)	PET-CT/MRI defined lesion	2.9 cc (IQR 1.7–5)	GTV + 5–7 mm constrained to prostate + regions with positive biopsies	9.5 cc (7.2–15)	CTV + 2 mm	16 cc (IQR 12.2–22.3)	PTV D95% ≥95% prescribed dose	D2% < 115% (optimal)	
Patel [30]	SBRT	40Gy/5# 75% 42.5Gy/5# 25%	Alternate days	Fiducials Spacer 25%	Focal	PET-CT/MRI defined	3.7 cc (0.4–11.2)	–	–	GTV + 5 mm (3 mm superiorly and posteriorly)	9.7 cc (3.3–25.9)	Prescription dose prescribed to 100% isodose	PTV V110% prescribed dose <5%	
Patel [29]	SBRT	40Gy/5# 33% 42.5Gy/5# 67% 30Gy/5# to whole prostate	Alternate days	Empty rectum Full bladder Fiducials Spacer 33%	Whole gland + SIB	PET-CT/MRI defined SIB	8.3 cc (0.2–16.4) for SIB	Prostate + − SVs for whole gland + GTV + 0 mm for SIB	–	PTV prostate CTV + 3–5 mm PTV SIB GTV + 5 mm (3 mm superiorly and posteriorly)	19 cc (1.3–32.9) for SIB	Prescription dose prescribed to 100% isodose	PTV V110% prescribed dose <5%	OAR constraints prioritised
														
Crook (including Crook 2019) [20]	LDR BT	140Gy I-125 92% 120Gy Pd-103 8%			Whole gland 98% Focal 2%			Prostate (TRUS)	TRUS volume 25 cc (14–44)	CTV + 2–3 mm anterolaterally and 5 mm superoinferiorly		PTV V100% ≥90%	PTV I-125 V150% <45% V200% <10% PTV Pd-103 V150% <55% V200% <15%	
Chitmanee [19]	HDR BT	19Gy/1#			Focal			MRI defined lesion (some cases also used PET-CT) + regions with positive biopsies	9.6 cc (2.4–37.5)	CTV + 3 mm constrained to rectum and urethra	18.6 cc (7.4–66.4)	CTV V100% ≥100% prescribed dose PTV V100% ≥95%		
Ménard [26]	HDR BT	Focal: 22-26Gy/2# Whole gland + focal SIB: 16-22Gy/2# + 22-26Gy SIB	5–14 days apart		Focal 83% Whole gland 17%	PET-CT/MRI defined lesion		Focal: GTV + 5 mm restricted to 2 mm beyond prostate and excluding urethra Whole gland: Prostate		CTV + 2 mm superoinferiorly		GTV V100% ≥99% prescribed dose PTV V100% ≥95%		OAR constraints prioritised
Nguyen [27]	LDR BT	137Gy/1#			Whole gland			Prostate				V100% ≥100% prescribed dose D90% to receive prescribed dose		
Paulin (update of Murgic 2018 and Corkum 2022) [32]	HDR BT	27Gy/2#	1–2 weeks apart		Focal	MRI defined lesion		GTV + 3–5 mm constrained to prostate and urethra PRV		CTV + 0 mm	4.8 cc (IQR 3.5–6.3)	PTV V100% ≥95% prescribed dose	V150% <65% V200% <35%	OAR constraints prioritised
Rasing (update of van Son 2020, van Son 2020 and Maenhout 2017) [33]	HDR BT	19Gy/1#			Focal	PET-CT/MRI defined lesion	2.9 cc (IQR 1.7–5.1)	GTV + 5 mm constrained to prostate	9.9 (IQR 7–14.5)	No PTV used		CTV D95% ≥100% prescribed dose D90% ≥17Gy		
Ryg [34]	HDR BT 70% SBRT 30%	HDR BT 30Gy/3# SBRT 25Gy/5# + 35Gy SIB (1 patient 25Gy/5# whole gland and 1 patient 30Gy/6# whole gland but no SIBs)	HDR BT 2 weeks apart SBRT alternate days	Fiducials for SBRT	HDR BT: Focal SBRT: Whole gland + SIB	PET-CT/MRI defined lesion		Prostate		CTV + 3 mm		V100% ≥90% prescribed dose (mandatory) V100% ≥95% (optimal) D90% ≥100% (mandatory)		
Strnad [37]	HDR BT 34% + hyperthermia PDR BT 76% + hyperthermia	HDR BT 30Gy/3# PDR BT 60Gy/2#	HDR BT 3 weeks apart PDR BT 3 weeks apart		Whole gland			Prostate		CTV + 0 mm		D90% >100% V100% >90–95%		
Yamada [35]	PDR BT	32Gy/4#	Minimum of 4 h apart over a total of 30 h		Whole gland		Pretreatment gland volume 33.5 cc (11–64)	Prostate +5 mm				V100% ≥95% prescribed dose		

BT, brachytherapy; CTV, clinical target volume; Dx% or cc, dose to x% or cc [of the volume]; GTV, gross tumour volume; HDR BT, high dose rate brachytherapy; IMRT, intensity-modulated radiotherapy; LDR BT, low dose rate brachytherapy; MRI, magnetic resonance imaging; OAR, organ at risk; PDR BT, pulsed dose rate brachytherapy; PET-CT, positron emission tomography-computed tomography; PRV, planning risk volume; PTV, planning target volume; reRT, reirradiation; RT, radiotherapy; SBRT, stereotactic body radiotherapy; SIB, simultaneous integrated boost; TRUS, transrectal ultrasound; Vx% or Gy, volume receiving x% [of the prescribed dose] or dose in Gy.

⁎ Majewski et al. study ongoing but published preliminary toxicity results from subset of cohort included here.

Dose-fractionation schedules for SBRT ranged from 25 to 42.5Gy in 5 fractions [18], [22], [23], [24], [25], [28], [29], [30], [34], [36] and 36-38Gy in 6 fractions [17], [18], [28], delivered on alternate days in most cases. For brachytherapy, 5 studies used HDR brachytherapy, ranging 19Gy in 1 fraction, 22-27Gy in 2 fractions or 30Gy in 3 fractions [19], [26], [32], [33], [34], and 2 studies used LDR brachytherapy, ranging 120-140Gy in 1 fraction (depending on isotope used) [20], [27]. One study used 38Gy in 4 fractions pulsed dose rate (PDR) brachytherapy and 1 used both PDR and HDR brachytherapy [35], [37]. Patient preparation for SBRT varied from no specific preparation in some studies through to combinations of full bladder, empty rectum and fiducial markers with/without use of rectal spacers or endorectal balloons.

Focal and whole gland target volumes (with/without simultaneous integrated boost, SIB) were used in 5 [17], [18], [28], [30], [36] and 4 [21], [23], [31], [34] SBRT studies, respectively, with the remainder containing patients treated using both approaches [22], [24], [25]. Four brachytherapy studies used whole gland target volumes [20], [27], [35], [37], 4 studies used focal target volumes [19], [32], [33], [34] and 1 study used both approaches [26].

Gross tumour volume (GTV) for focal treatments or SIBs was generally defined using PET-CT and MRI. Clinical target volume (CTV) for focal treatments/SIB encompassed GTV with 0-7 mm margins, usually constrained to prostate and, in some cases, Urethra or Urethra planning risk volume (Urethra_PRV). CTV was prostate with/without extra-prostatic extension for studies which used whole gland approaches. Most SBRT and some brachytherapy studies used a planning target volume (PTV) which ranged from 2 to 5 mm from CTV, with some studies stipulating a smaller margin posteriorly or adjacent to urethra.

In most SBRT studies, target volume objectives ranged from 95 to 100% of prescription dose to 95–100% of PTV. Most studies permitted 2–5% of PTV to receive up to 107–110% of prescription dose with 2 studies aiming for up to 130–150% of prescription dose within GTV [17], [23]. In brachytherapy studies, target volume objectives ranged from 90 to 100% of prescription dose to 90–100% of PTV.

#### 3.1.3 Organs at risk definitions, constraints and reported doses

OAR definitions, constraints and reported doses are summarised in Table 3. OAR constraints were reported in physical doses and applied to the reirradiation treatment only- no study reported use of cumulative constraints which accounted for previously delivered dose. OARs are described as per American Association of Physicists in Medicine TG263 nomenclature [38].

**Table 3:** Organ at risk definitions, constraints, planned doses and toxicities in prospective published studies.

Study	ReRT technique	Bladder definition	Constraint	Planned doses	Urethra definition	Constraint	Planned doses	Acute GU toxicity (CTCAE unless otherwise stated)	Late GU toxicity (CTCAE unless otherwise stated)	Rectum definition	Constraint	Planned doses	Acute GI toxicity (CTCAE unless otherwise stated)	Late GI toxicity (CTCAE unless otherwise stated)	QoL
Bergamin [17]	SBRT (36-38Gy/6#)	Bladder	D0.1cc ≤33Gy D0.5cc ≤28 Gy D1cc ≤24Gy D2cc ≤ 18Gy	D1cc 11.9Gy (2–30.9)	Urethra_PRV (2 mm)	Urethra Dmax <33Gy Urethra PRV Dmax <36Gy	0 (0.0–30.1)	RTOG G1 24% G2 4%	RTOG G1 28% G2 4%	Rectum	D0.1cc ≤33Gy D0.5cc ≤28 Gy D1cc ≤24Gy D2cc ≤ 18Gy	D1cc 16.9 (11.8–28.8) Mean dose to whole rectum 5.7 (2.9–11.4)	RTOG G1 8% G3 4% (rectal ulcer/spacer)	RTOG G1 8%	
Buchalet (including Michalet 2022) [18]	SBRT (30Gy/5# or 36Gy/6#)	Bladder_Wall	V27Gy <5 cc V12Gy <15%	–	Urethra_PRV (3 mm)	Dmax (0.035 cc) <39Gy V36Gy <1 cc V24Gy <30%		G1 29.2% G2 8.5% G3 1.9%	G1 24.7% G2 4.5% G3 3.4%	Rectal_Wall	V27Gy <2 cc V12Gy <20%	–	G1 7.5%	G1 6.7%	
Cuccia [21]	SBRT (30Gy/5#)	Bladder	V10Gy <25% V18Gy <15%	–	Urethra	Dmax <30Gy	–	G1 50% G2 18.2%	G1 50% G2 18.2%	Rectum	V10Gy <40% V18Gy <20%	–	G1 31.8% G2 18.2%	G1 27.3% G2 13.6%	
Ekanger [22]	IMRT (35Gy/5#)	–	None applied	–	–	None applied	–	RTOG G1 39% G2 18% G3 5%	RTOG G1 37% G2 26% G3 16% (1 patient cystoprostatectomy/rectal surgery, 1 patient permanent SPC, 5 patients HBOT)	Rectum	Dmax 25Gy (EQ.D2)	–	RTOG G1 26% G2 5%	RTOG G1 16% G2 8% G3 3%	
Fuller [23]	SBRT (34Gy/5#)	Bladder_Wall	Dmax <34Gy	–	Urethra	Dmax 40.8Gy D50% 35.7Gy	–	No G3+	G2+ 17% G3+ 8%	Rectal_Wall Rectal mucosa	Dmax <34Gy Dmax <25.5Gy	–	No G3+	No G2+	IPSS increased by median 5 points up to 1 year post SBRT, returned to baseline by 3 years
Greco [24]	SBRT (35-40Gy/5#)	Bladder	D2% ≤35Gy D1cc ≤35Gy D50% ≤17.5Gy	D2% 32.1Gy (IQR 28.8–32.6) D1cc 33.1Gy (IQR 32.1–33.9) D50% 4.6Gy (IQR 2.0–10.5)	Urethra_Wall (2 mm expansion around catheter)	D2% ≤31.5Gy D1cc ≤28Gy	D2% 30.2Gy (IQR 29.9–35.0) D1cc 26.2Gy (IQR 24.8–26.5)	G1 40% G2 20%	G1 36.7% G2 13.3%	Rectal_Wall	D2% ≤35Gy, D1cc ≤31.5Gy D50% ≤21Gy	D2% 30.9Gy (IQR 30.6–31.4) D1cc 30.7Gy (IRQ 30.0–31.0) D50% 7.9Gy (IQR 4.9–9.4)	G1 20%	G1 20%	Change in urinary QoL at 1 month (IPSS increased median 4 points, *p* = 0.2 and EPIC-26 decreased median 8.5 points, *p* = 0.018), return to baseline 3 months, further change 6–12 months with return to baseline again by 18 months No significant change in EPIC-26 bowel domains
Lewin [25]	SBRT (30–40Gy/5#)	–	–	–	Urethra_PRV	Dmax <32.5Gy		G2 27% G3 3% (1 patient haematuria)	G2 30% G3 3.3% (1 patient urethral stricture)	Rectal_Wall	V100% <5% V90% < 15% V80% < 20% (of prescribed prostate dose) V38Gy <2 cc		No G2+ toxicity	G2 3% (incontinence)	
Majewski⁎# [36]	SBRT 33.75Gy/5#	Bladder	Dmax ≤35.4Gy D30% <15Gy		Urethra	Dmax ≤35.4Gy D30% <15Gy		G1 47.6% G3 4.8% (1 patient haematuria)	G1 23.8% G2 23.8% G3 4.8% (1 patient haematuria)	Rectum	Dmax ≤34.8Gy D30% <15Gy		G1 14.2% G2 9.5%	G1 19% G2 4.8%	
Pasquier [28]	SBRT (25-30Gy/5# or 36Gy/6#)	Bladder_Wall	V27Gy <5 cc V12Gy <15%	Median Dnear-max 12.4Gy (IQR 8.5–18.5) V12Gy median 2.1% (IQR 0.4–7.1) V27Gy median 0 cc (IQR 0–0.3)	Urethra_PRV (3 mm)	D0.035cc <39Gy V24Gy <30% V36Gy <1 cc	D0.035ccmedian 36.6Gy (IQR 31.9–37.3) V24Gy median 29.1% (IQR 23.2–31) V36Gy median 0.2 cc (IQR 0–0.5)	G2 19%	G2 33%	Rectal_Wall	V27Gy <2 cc V12Gy <20%	Dnear-max median 26.4Gy (IQR 22.1–30.2) V12Gy median 15.6% (IQR 10.9–18.4) V27Gy median 0.6 cc (IQR 0.1–1.1)	No G2+ toxicity	No G2+ toxicity	
Patel [30]	SBRT (40–42.5Gy/5#)	Bladder_Wall	40Gy: Dmax <42Gy 42.5Gy: Dmax <44.6Gy	40Gy: Dmax 17.1Gy (5.1–39) D1cc 9.4Gy (1.9–23.5) 42.5Gy: Dmax 40.1Gy (36.7–45.1) D1cc 33.4Gy (23–43.9)	Urethra	40Gy: Dmax <42Gy 42.5Gy: Dmax <44.6Gy	40Gy: Dmax 40.3Gy (24.5–40.7) 42.5Gy: Dmax 43.5Gy (43.3–43.8)		Acute/late toxicity 40Gy: G1 100% G2 50% 42.5Gy: G2 100% G3 100% (1× haematuria, 1× incontinence)	Rectal_Wall (outside PTV)	40Gy: Dmax <40Gy 42.5Gy: Dmax <42.5Gy	40Gy: Dmax 39 (23.9–41.8) D1cc 26.1 (15.5–31.2) 42.5Gy: Dmax 35.8 (29.1–42.4) D1cc 17.5 (12.8–22.3)		Acute/late toxicity 40Gy: G1 33.3% G2 16.7% (both had rectal spacer) 42.5Gy: No G1+ toxicity	Change in urinary QoL from baseline 3–18 months (AUA, EPIC-26 irritation and EPIC-26 incontinence scores, *p* < 0.05) No significant change in EPIC-26 bowel domains
Patel [29]	SBRT (SIB 40–42.5Gy/5#, whole prostate 30Gy/5#)	Bladder_Wall	40Gy: Dmax <42Gy 42.5Gy: Dmax <44.6Gy	40Gy: Dmax 35.3(IQR 34.9–36) D1cc 35.3 (IQR 34.9–36) 42.5Gy: Dmax 34.9 (IQR 34–35.5) D1cc 34.9 (IQR 34–35.5)	Urethra	40Gy: Dmax <42Gy 42.5Gy: Dmax <44.6Gy	40Gy: Dmax 41.2Gy (IQR 40.7–41.3) 42.5Gy: Dmax 43.1Gy (IQR 42.7–43.5)		40Gy: G2 100% 42.5Gy: G2 100%	Rectal_Wall (outside prostate PTV)	40Gy: Dmax <40Gy 42.5Gy: Dmax <42.5Gy	40Gy: Dmax 32.6 (IQR 32–33.9) D1cc 25.7 (IQR 25.2–26.6) 42.5Gy: Dmax 40.3 (IQR 38.8–42.6) D1cc 30.5 (IQR 26.6–33.8)		40Gy: G1 33% G2 67% 42.5Gy: G1 17% G2 17%	Change in urinary QoL from baseline 3–18 months (AUA, EPIC-26 irritation and EPIC-26 incontinence scores, p < 0.05) No significant change in EPIC-26 bowel domains
															
Crook (including Crook 2019) [20]	LDR BT (140Gy/1# I-125 or 120Gy/1# Pd-103)								Acute/late toxicity G3 GU/GI 14% (frequency/incontinence, fistula, cystitis, urinary obstruction, urethral stricture, proctitis, rectal pain, rectal bleeding)					Acute/late toxicity G3 GU/GI 14% (frequency/incontinence, fistula, cystitis, urinary obstruction, urethral stricture, proctitis, rectal pain, rectal bleeding)	
Chitmanee [19]	HDR BT (19Gy/1#)				Urethra	D10% <22Gy D30% <20.8Gy	D10% 15.5Gy (5.5–26.2) D30% 18.5Gy (6.8–28.8)	G1 36% G2 54%	G1 26% G2 46% G3 10% (haematuria, obstruction, urethral stricture)	Rectum	D2cc <15Gy	D2cc 9Gy (1.9–14.9)	G1 24% G2 8%	G1 22% G2 8%	
Ménard [26]	HDR BT (22-26Gy/2#)				Urethra	Dmax <32Gy D0.5cc <26Gy			Focal: G1 33% G2 15% Whole gland: G1 33% G2 60% *p* < 0.01	Rectum	D0.5cc <20Gy			Focal: G1 5% Whole gland: G1 27% G2 20% *p* < 0.01	Urinary QoL (EPIC) returned to baseline by 3 months Bowel QoL (EPIC) returned to baseline by 3 months
Nguyen [27]	LDR BT (137Gy/1#)				Urethra				RTOG G3 8% (1 patient prostatic abscess treated with drainage and 1 patient urethral stricture) G4 12% (3 x prostate-rectal fistula)	Anterior Rectal_Wall				RTOG G3 8% (2 patients rectal bleeding) G4 12% (3 x prostate-rectal fistula)	
Paulin (update of Murgic 2018 and Corkum 2022) [32]	HDR BT (27Gy/2#)				Urethra_PRV (1 mm)	D10% <29.7Gy (110% prescribed dose)	D10% 68.8% prescribed dose (13.6–115.5)	No G3+	Prevalence G2+ 24 months 11.9% 48 months 13.6% (1 patient G3 cystitis)	Rectum	V80% ≤0.5 cc	V80 0 cc (0–0.6)	No G3+	Prevalence G2+ 24 months 1.7% 48 months 0%	No significant change in IPSS scores EPIC-50 urinary domain changed at 1 month (*p* = 0.001) but no differences at other timepoints No significant change in EPIC-50 bowel domains
Rasing# (update of van Son 2020, van Son 2020 and Maenhout 2017) [33]	HDR BT (19Gy/1#)	Bladder	D1cc <12Gy	D1cc 9.2Gy (IQR 5.2–11.6)	Urethra	D10% <17.7Gy	D10% 15.2Gy (IQR 10.3–17.5)	G1 45.8% G2 20.8%	G1 18.9% G2 40.9% G3 3.9% (1 patient G3 cystitis, 2 x urethral stricture, 2× incontinence)	Rectum	D1cc <12Gy	D1cc 10.2Gy (IQR 8.1–11.7)	G1 20.1% G2 2.1%	G1 25.2% G2 4.7%	EORTC QLQ-PR25 increased by 11 points at 1 month but returned to baseline No clinically meaningful change in EORTC QLQ-PR25
Ryg [34]	HDR BT (30Gy/3#) SBRT (25-35Gy/5# or 30Gy/6#)	Bladder	SBRT: D2cc <25 Gy (mandatory) D0.05cc <25 Gy (optimal) D15cc <18.3Gy		Urethra_PRV (3 mm)	HDR BT: D0.1cc <5Gy SBRT: Urethra D0.1cc <25 Gy Urethra PRV D0.1cc <30 Gy D0.05cc <25 Gy	D0.1cc 29.1Gy (SD 8.2)	RTOG G1 42.4% G2 6.1% G3 3%	RTOG G1 24.2% G2 12.1%	Rectum	HDR BT: D2cc <12Gy SBRT: D0.5cc <25 Gy D30% <14 Gy	D2cc 17Gy (SD 7.9)	RTOG G1 18.2%	RTOG G1 24.2% G2 3%	
Strnad [37]	HDR BT 34% + hyper-thermia PDR BT 76% + hyper-thermia				Urethra	D0.1cc <120% prescribed dose			G1–2 20% G3 8% (incontinence)	Rectum	D2cc <50Gy (EQ.D2)			G1 < 1%	IPSS improved from severe (30–35 points) to moderate (10–15 points) post-treatment
Yamada [35]	PDR BT (32Gy/4#)				Urethra	Dmax <38.4Gy (120% prescribed dose)	Dmax 116% prescribed dose (110–127) D5% 113% (104–122) D20% 110% (100–119)	G1 38% G2 40%	G1 38% G2 48% G3 9.5% (1 patient incontinence)	Rectum	Dmax <32Gy (100% prescribed dose)	V100% median 0 cc (0–0.1) D1cc 61.5% prescribed dose (18–86) D2cc 54% (14–79)		G1 43% G2 14%	IPSS increased by median 6 points at last follow up

AUA, American Urological Association; BT, brachytherapy; Dx% or cc, dose to x% or cc [of the volume]; CTCAE, Common Terminology Criteria for Adverse Events; Dmax, maximum dose [to the volume]; EPIC, Expanded Prostate cancer Index Composite; EQ.D2, equivalent dose in 2Gy fractions; HDR BT, high dose rate brachytherapy; IMRT, intensity-modulated radiotherapy; IPSS, International Prostate Symptoms Score; LDR BT, low dose rate brachytherapy; MRI, magnetic resonance imaging; PDR BT, pulsed dose rate brachytherapy; PET-CT, positron emission tomography-computed tomography; PRV, planning risk volume; QoL, quality of life; reRT, reirradiation; RTOG, Radiation Therapy Oncology Group; SBRT, stereotactic body radiotherapy; SIB, simultaneous integrated boost; Vx% or Gy, volume receiving x% [of the prescribed dose] or dose in Gy.

⁎ Majewski et al. study ongoing but published preliminary toxicity results from subset of cohort included here.

# Toxicity data reported from van Son et al., 2020, QoL data reported from van Son et al., 2020.

##### 3.1.3.1 Bladder

Bladder (4 SBRT [17], [21], [24], [36] and 2 brachytherapy [33], [34] studies) and Bladder_Wall (5 SBRT studies [18], [23], [28], [29], [30]) structures were used as the OAR. Bladder OAR constraints varied from near-maximum doses (e.g. maximum dose, Dmax, or dose to 0.1 cc, D0.1cc, often close to the prescribed dose) to larger volume constraints (e.g. dose to 15% of the Bladder/Bladder_Wall). Five SBRT studies reported median planned Bladder/Bladder_Wall doses[17, 24, 28, 30, 31]- in all cases, these met the specified constraints aside from in Patel et al. [30], where in some cases the Dmax was exceeded.

In two HDR brachytherapy studies, constraints of 12Gy in 1 fraction to 1 cc Bladder and 25Gy in 3 fractions to 2 cc Bladder were used [33], [34]. The remaining brachytherapy studies did not use bladder constraints.

##### 3.1.3.2 Urethra

Urethra and Urethra_PRV (2–3 mm) structures were used in 5 [21], [23], [30], [31], [36] and 5 [17], [18], [24], [25], [28] SBRT studies, respectively. In focal and whole gland SBRT studies, near-maximum Urethra/Urethra_PRV constraints ranged from 25 to 44.6Gy in 5 fractions [21], [23], [25], [29], [30], [34], [36] and 39Gy in 6 fractions [18], [28], respectively. One SBRT study also used a volumetric Urethra constraint of D30% <15Gy [36]. Of 5 studies which reported median planned Urethra/Urethra_PRV doses [17], [24], [28], [29], [30], median and/or highest reported doses exceeded constraints in three studies [28], [29], [30].

Six HDR brachytherapy studies used Urethra or Urethra_PRV (1–3 mm) constraints, which were heterogenous and included near-maximum and larger volume limits [19], [26], [32], [33], [34], [35], [37]. In 3 brachytherapy studies which described planned Urethra/Urethra_PRV doses, median doses were within tolerance but highest reported doses appear to have exceeded constraints [19], [32], [35].

##### 3.1.3.3 Rectum

A Rectum structure was used in 4 SBRT [17], [21], [22], [36] and 7 brachytherapy [19], [26], [32], [33], [34], [35], [37] studies, respectively, while 7 SBRT studies used a Rectal_Wall structure [18], [23], [24], [25], [28], [29], [30] and 1 study also used rectal mucosa [23]. In focal and whole gland SBRT studies, near-maximum constraints of 25–42.5Gy in 5 fractions [23], [29], [30], [34], [36] and 33Gy in 6 fractions [17] were used. Two focal SBRT studies used volumetric constraints of 12Gy in 5–6 fractions to 20% of Rectal_Wall [18], [28]. One SBRT study also used a volumetric Rectum constraint of D30% <15Gy [36]. Of 5 SBRT studies which reported planned rectal/Rectal_Wall doses [17], [24], [28], [29], [30], constraints were met in 3 [17], [24], [28]. In Patel et al. and Patel et al., median doses were within tolerance but the highest reported Dmax exceeded constraints [29], [30].

Six HDR brachytherapy studies used a variety of near-maximum and small volume rectal constraints [19], [26], [32], [33], [34], [37]. Planned doses did not appear to exceed constraints in 3 studies which reported these [19], [32], [35].

#### 3.1.4 Toxicity and quality of life

GU and GI toxicity and quality of life outcomes are summarised in Table 3. Toxicity assessment used Common Terminology Criteria for Adverse Events (CTCAE) except in 4 studies which used Radiation Therapy Oncology Group (RTOG) [17], [22], [27], [34]. There was variation in median follow up duration, which ranged 8–81 months and 20–81 months for SBRT and brachytherapy studies, respectively. The short follow-up of some series does not allow precise quantification of the incidence of late toxicities.

##### 3.1.4.1 Genitourinary toxicity and quality of life

In focal SBRT studies, acute GU toxicity rates ranged from 24 to 47.6%, 4–19% and 1.9–4.8% for grades 1, 2 and 3, respectively [17], [18], [28], [36]. Late GU toxicity rates ranged from 4 to 23.8%, 3.7–37% and 2.8–4.8% for grades 1, 2 and 3, respectively [17], [18], [28], [36]. In the study by Patel et al., grade 3 toxicity (incontinence and haematuria) was observed in both patients treated using 42.5Gy in 5 fractions [30].

In SBRT studies which used whole gland and/or focal SBRT, acute GU toxicity rates ranged from 40 to 50%, 18.2–26% and 5–3.3% for grades 1, 2 and 3, respectively [21], [22], [24], [25]. Late GU toxicity rates ranged from 36.7 to 50% and 13.3–100% for grades 1 and 2, respectively [21], [22], [23], [24], [25], [29]. The late grade 2 GU toxicity rate of 100% occurred in all 9 participants in Patel et al. [29]. Late grade 3 GU toxicities were observed in three studies: 8% of patients in Fuller et al. [23] and 16% of patients in Ekanger et al. [22] and 3.3% of the patients in Lewin et al. [25]. Across these three studies, one did not specify bladder or urethral constraints, while one permitted a relatively high maximum dose (150%) within the PTV.

Four SBRT studies described participant-reported GU quality of life (QoL) [23], [24], [29], [30], [31]. Of these two described an initial worsening of symptom scores but a return to baseline over the longer (18 months to 3 years) term. Patel et al. noted change in American Urological Association (AUA) and Expanded Prostate Cancer Index Composite (EPIC)-26 irritative scores and EPIC-26 incontinence scores from 3 to 18 months across two phase 1 studies [29], [30], [31].

In focal brachytherapy studies, acute GU toxicity rates were 36% and 54% for grades 1 and 2, respectively, in Chitmanee et al. [19]. Late GU toxicity rates in focal brachytherapy studies ranged from 11.9 to 33%, 13.6–46% and 1.7–10% for grades 1, 2 and 3, respectively [19], [26], [32]. Grade 3 toxicity events included haematuria, obstruction and urethral stricture in Chitmanee et al. and cystitis in Paulin et al. [19], [32]. In both studies, median dose to 10% of Urethra/Urethra_PRV was within constraints but highest reported planned doses exceeded these.

In studies of whole gland brachytherapy, late GU toxicity rates ranged from 24.2 to 38%, 12.1–60%, 8–14% and 12% for grades 1, 2, 3 and 4, respectively [20], [26], [27], [35], [37]. Grade 3 events included frequency/incontinence, fistula, cystitis, urinary obstruction, urethral stricture and prostatic abscess [20], [27], [37]. In two of these studies, prescription doses (137-140Gy using I-125) were similar to those used for primary LDR brachytherapy while neither study described bladder/urethra constraints [20], [27]. Interstitial hyperthermia was used alongside HDR/PDR brachytherapy in the other study [37].

Three brachytherapy studies described GU QoL [26], [32], [37]. Ménard et al. observed EPIC urinary QoL return to baseline by 3 months post-treatment [26]. Paulin noted no significant change in International Prostate Symptoms Score (IPSS) scores and change in EPIC-50 urinary at 1 month but no difference at later timepoints [32]. Strnad et al. observed an improvement in IPSS scores from severe (30–35 points) at baseline to moderate (10–15 points) post-brachytherapy [37].

##### 3.1.4.2 Gastrointestinal toxicity and quality of life

In focal SBRT studies, acute GI toxicity rates ranged from 7.4 to 14.2% for grade 1 and 9.5% for grade 2 [17], [18], [36]. Four percent grade 3 toxicity was observed in Bergamin et al., in a patient with rectal spacer inserted pre-SBRT [17]. Late GI toxicity rates ranged from 5.6 to 33.3% for grade 1 and 4.8–16.7% for grade 2 [17], [18], [30], [36]. In Patel et al., grade 2 events of 16.7% were observed in 2 patients with rectal spacers [30].

In SBRT studies which used whole gland and/or focal SBRT, acute GI toxicity rates ranged from 20 to 31.8% and 0–18.2% for grades 1 and 2, respectively [21], [22], [24]. Late GI toxicity rates ranged from 17 to 33%, 3–67% and 3% for grades 1, 2 and 3, respectively [21], [22], [24], [29].

Two studies describe participant-reported GI QoL, with neither Greco et al. nor the Patel et al. studies observing significant changes in EPIC-26 bowel domain scores [24], [29], [30], [31].

In terms of focal brachytherapy studies, acute GI toxicity rates of 24% and 8% were observed for grades 1 and 2, respectively in one study [19]. Late GI toxicity rates ranged from 5 to 22% and 1.7–8% for grades 1 and 2, respectively across three studies [19], [26], [32].

In focal and/or whole gland brachytherapy studies, late GI toxicity rates of 1–43%, 14–20% and 8–14% for grades 1, 2 and 3, respectively [20], [26], [27], [35], [37]. Grade 3 events included proctitis, rectal pain and rectal bleeding [20], [27]. Grade 4 events were reported in one study where 12% (*n* = 3) developed grade 4 prostate-rectal fistulae [27]. As above, prescription doses were similar to the primary brachytherapy setting and neither study described OAR constraints.

Two brachytherapy studies describe GI QoL, with Paulin et al. observing no significant changes in EPIC-50 bowel domain scores and Ménard et al. observed EPIC bowel scores return to baseline by 3 months post-treatment [26], [32].

### 3.2 Ongoing prospective studies

Patient, disease and treatment characteristics for ongoing studies are summarised in Table 4.

**Table 4:** Patient, disease and treatment characteristics for ongoing prospective studies.

Study	Type	Number of participants	Type of reRT	Initial RT technique	Staging at reRT	ISUP score at reRT	PSA at reRT	PSAdt at reRT	Imaging for local recurrence	Biopsy confirmation of local recurrence	Time interval to reRT	Pre-existing toxicity	Urinary function reRT	ADT with reRT
Henry (NCT05614700)	Randomised feasibility	60	SBRT vs HDR BT	EBRT and/or BT	T1–3	Any	<50		PET-CT MRI	Yes	Minimum 2 years		IPSS <20 Qmax >10	Clinician discretion
sss	Phase 1	36	SBRT	EBRT	T1–2a		<10		PET-CT MRI	Yes	Minimum 4 years (5 years if previous ADT)	No G3+ toxicity		No
Kang (ACTRN12625000224426)	Phase 2	10	SBRT	EBRT and/or BT	Any T	Any			PET-CT MRI	Yes	Minimum 2 years		IPSS <20	6 months
Majewski (NCT06201078)	Phase 2	55	SBRT	EBRT					PET-CT MRI	Not mandated	Minimum 2 years	No G2+ or prior G3+ toxicity	IPSS <19	Not recommended
Pasquier (NCT03438552)	Phase 2	57	SBRT	EBRT	T1–3a (not posteriorly)		≤10	>10 months	PET-CT MRI	Yes	Minimum 2 years	No G2+ toxicity	IPSS ≤15	No
Taggar (NCT05597852)	Phase 1/2	10	SBRT	EBRT (not SBRT with ≥5Gy/#)	Any T	Any			PET-CT MRI	Yes			IPSS <20	6 months
														
Burchardt (NCT05715502)	Phase 2	100	LDR or HDR BT	EBRT and/or BT	T1–2		≤10	>6 months	PET-CT MRI	Yes	Minimum 2 years	‘Low’ toxicity after previous EBRT and/or BT	IPSS <20	No
Crook (NCT03246802)	Prospective cohort	30	HDR BT	EBRT and/or BT	T1–2		≤10	>6 months	MRI	Yes	Minimum 3 years	No G2+ toxicity	IPSS <16 or Qmax >10 and PVR <100	Clinician discretion
Dimnik (NCT06202248)	Feasibility	10	DaRT seeds	EBRT and/or BT	T1–2, lesion <3 cm		≤10		PET-CT MRI	Yes				
DiNome (NCT02899221)	Phase 1	3	HDR BT + hyperthermia	EBRT and/or BT					MRI CT	Yes		No prior surgical procedures predisposing to GU toxicity		
Solanki (NCT03312972)	Phase 1/2	62	HDR BT	EBRT and/or BT	T1–3b				PET-CT MRI	Yes		No G3+ toxicity	IPSS <20	Clinician discretion

ADT, androgen deprivation therapy; BT, brachytherapy; CT, computed tomography; EBRT, external beam radiotherapy; DaRT, radium-224; HDR BT, high dose rate brachytherapy; HIFU, high frequency ultrasound; IPSS, International Prostate Symptom Score; IQR, inter-quartile range; ISUP, International Society of Urological Pathologists; LDR BT, low dose rate brachytherapy; MRI, magnetic resonance imaging; PDR BT, pulsed dose rate brachytherapy; PET-CT, positron emission tomography-computed tomography; PORT, post-operative radiotherapy; PSA, prostate specific antigen; PSAdt, prostate specific antigen doubling time; reRT, reirradiation; SBRT, stereotactic body radiotherapy.

#### 3.2.1 Summary of ongoing studies

In total 11 ongoing studies were identified: Five using SBRT, 5 using LDR or HDR brachytherapy and one using both SBRT and HDR brachytherapy, including phase 1 and 2 studies with 1 randomised feasibility study. No phase 3 studies were identified.

Anticipated number of patients per study ranges from 3 to 100. Almost all studies require staging with both PET-CT and MRI. Biopsy confirmation of local recurrence is mandated in all but 1 SBRT study.

Most SBRT studies permit T1–3 disease at reirradiation whereas most brachytherapy studies restrict this to T1–2. Five studies require PSA ≤10 ng/ml with 1 study using a threshold of ≤50 ng/ml. Three studies require PSA doubling time > 6–10 months.

Minimum time interval between initial radiotherapy and reirradiation ranges from 2 to 4 years. Most studies stipulate no pre-existing GU or GI toxicity, but whether this is a grade ≥ 2 or ≥ 3 threshold varies. Some studies specify an IPSS cut-off (most used an IPSS <20), and some include formal urinary flow assessment with peak of >10 ml/s as eligibility criteria. There is varying use of ADT with reirradiation.

#### 3.2.2 Technical characteristics

Technical characteristics are summarised in Table 5. Limited data is available for the brachytherapy studies.

**Table 5:** Reirradiation technical characteristics for ongoing prospective studies.

Study	ReRT technique	ReRT dose-fractionation	Timing	Preparation	Target	GTV definition	CTV definition	PTV definition	Prescription point	Maximum dose	Prioritisation
Henry (NCT05614700)	SBRT or HDR BT	SBRT: 36.25Gy/5# HDR BT: 27Gy/2#	SBRT: Alternate days HDR BT: 2 weeks apart	Clinician discretion	Focal or whole gland	PET-CT/MRI defined lesion	GTV + 3 mm constrained to prostate (and urethra and rectum for HDR BT)	SBRT: CTV + 3–5 mm HDR BT: CTV + 0 mm	SBRT: GTV V40Gy ≥95% prescribed dose PTV V36.25Gy ≥85–95% HDR BT: GTV V100% prescribed dose ≥90% PTV D90% ≥24.2Gy	SBRT: GTV ≤130% prescribed dose HDR BT: GTV V150% prescribed dose <45% GTV V200% <15%	
Hruby (NCT03073278)	SBRT	36Gy/6# 38Gy/6#	Alternate days	Full bladder Empty rectum Fiducials Spacer if lesion adjacent to rectum	Focal	PET-CT/MRI defined lesion	GTV + 3 mm constrained to prostate/urethra	CTV + 3 mm	PTV D95% ≥100% prescribed dose CTV D95% ≥100% GTV D100% ≥100%	GTV D0.1cc ≤130% PTV D0.1cc ≤115%	
Kang (ACTRN12625000224426)	SBRT	25Gy in 5# + SIB 35Gy	Weekly	Empty rectum Full bladder Fiducials Spacer	Whole gland + SIB	PET-CT/MRI defined lesion	Whole gland: Prostate +1 cm SV	PTV tumour: GTV + 4 mm PTV whole gland: CTV + 4 mm	SIB: GTV V35Gy ≥99% PTV V33.25Gy ≥99% Whole gland: CTV V25Gy ≥99% PTV V23.75Gy ≥99%	SIB: PTV V36.75Gy <5% V38.5Gy <1%	OAR constraints prioritised
Majewski (NCT06201078)	SBRT	33.75Gy/5#	Alternate days	Empty rectum Partially full bladder	Focal	PET-CT/MRI defined lesion	GTV + 1–3 mm	CTV + 3 mm (may be reduced to 1 mm in specific cases)			
Pasquier (NCT3438552)	SBRT	36Gy/6#	Alternate days	Empty rectum Full bladder Fiducials	Focal (CTV <50% prostate by volume)	PET-CT/MRI defined lesion	GTV + 5–7 mm constrained to prostate + regions with positive biopsies	CTV + 2 mm	PTV D95% ≥95% prescribed dose	D2% <115% (optimal)	
Taggar (NCT05597852)	SBRT	25Gy in 5# + SIB 35Gy	Weekly	Empty rectum Full bladder Fiducials Spacer	Whole gland + SIB	PET-CT/MRI defined lesion	Whole gland: Prostate +1 cm SV	PTV tumour: GTV + 4 mm PTV whole gland: CTV + 4 mm	SIB: GTV V35Gy ≥99% PTV V33.25Gy ≥99% Whole gland: CTV V25Gy ≥99% PTV V23.75Gy ≥99%	SIB: PTV V36.75Gy <5% V38.5Gy <1%	OAR constraints prioritised
											
Burchardt (NCT05715502)	LDR or HDR BT	LDR BT: 145Gy HDR BT: 26Gy/2#	HDR BT: 3 days-2 weeks apart		DIL	MRI and/or PET-CT	DIL + 5–10 mm margin within prostate +0–3 mm margin beyond capsule CTV must be ≤50% prostate volume		CTV V100% ≥95% D90 ≥ 100%	HDR BT: CTV V150% <50% V200% <20%	
Crook (NCT03246802)	HDR BT	HDR BT in 2#	Fractions 2 weeks apart		Focal	MRI		Ideally PTV volume < 65% of prostate volume			
Dimnik (NCT06202248)	DaRT seeds (Alpha radiation emitters)				Focal	PET-CT or MRI					
DiNome (NCT02899221)	HDR BT + hyperthermia										
Solanki (NCT03312972)	HDR BT	Up to 30Gy in 1–2#	Fractions 1–2 weeks apart		Focal	PET-CT/MRI defined lesion	GTV + 5–10 mm, restricted to exclude OARs	CTV + 0 mm	GTV & CTV D90% ≥100% prescribed dose GTV & CTV V100% ≥95%		OAR constraints prioritised

BT, brachytherapy; CTV, clinical target volume; Dx% or cc, dose to x% or cc [of the volume]; DIL, dominant intra-prostatic lesion; EBRT, external beam radiotherapy; GTV, gross tumour volume; HDR BT, high dose rate brachytherapy; IMRT, intensity-modulated radiotherapy; LDR BT, low dose rate brachytherapy; MRI, magnetic resonance imaging; OAR, organ at risk; PDR BT, pulsed dose rate brachytherapy; PET-CT, positron emission tomography-computed tomography; PRV, planning risk volume; PTV, planning target volume; reRT, reirradiation; SBRT, stereotactic body radiotherapy; SIB, simultaneous integrated boost; SV, seminal vesicles; Vx% or Gy, volume receiving x% [of the prescribed dose] or dose in Gy.

Dose-fractionation schedules for SBRT range from 33.75 to 36.25Gy in 5 fractions and 36-38Gy in 6 fractions, delivered on alternate days in all cases. For brachytherapy, 3 studies use HDR brachytherapy in 1–2 fractions, 1 study uses LDR or HDR brachytherapy and 1 study uses radium-224 seeds. Where described, patient preparation for SBRT varies from full bladder and empty rectum at clinician discretion through to addition of fiducial markers with/without use of rectal spacers, urethral catheter or endorectal balloons.

Three SBRT studies use a focal target volume and 3 use focal or whole gland with/without SIB approaches. In HDR brachytherapy studies which describe this, both focal and whole gland or focal target volume approaches are used.

GTV for focal treatments or SIBs is defined using PET-CT and MRI. For SBRT, CTV for focal treatments/SIB encompasses GTV with 1-7 mm margin, usually constrained to prostate. CTV is prostate for studies which use whole gland approaches. Two studies include proximal 1 cm of seminal vesicle in CTV. In the RO-PIP randomised feasibility study of SBRT vs HDR brachytherapy (NCT05614700) by Henry et al. and the F-SHARP HDR brachytherapy study (NCT03312972) by Solanki et al., no margin is used from CTV to PTV for brachytherapy [39], [40].

In SBRT studies, target volume objectives range from 95 to 100% of prescription dose to 85–100% of PTV. For SBRT, the RO-PIP study aims for 40Gy to GTV and 36.25Gy to PTV. Up to 130% of prescription dose within GTV is permitted in RO-PIP and Hruby (NCT03073278) studies with the Kang (ACTRN12625000224426) and Tagger (NCT05597852) studies mandating lower maximum doses within PTV.

#### 3.2.3 Organs at risk definitions and constraints

OAR definitions, constraints and reported doses are summarised in Table 6. Limited data is available for the brachytherapy studies. OAR constraints are reported in physical doses and apply to the reirradiation treatment only, except in the RO-PIP study which utilises cumulative constraints for SABR which account for previously delivered external beam radiotherapy dose to Bladder and Rectum.

**Table 6:** Organ at risk definitions, constraints, planned doses and toxicities for ongoing prospective studies.

Study	ReRT technique	ReRT dose-fractionation	Bladder definition	Constraint	Urethra definition	Constraint	Rectum definition	Constraint	Bowel	Constraint	Primary endpoint	Key secondary endpoints
Henry (NCT05614700)	SBRT or HDR BT	SBRT: 36.25Gy/5# HDR BT: 27Gy/2#	Bladder	SBRT: V27Gy <15 cc mandatory (10 cc optimal)	Urethra	HDR BT: Focal D10% <24.2Gy Whole gland D10% <20.2Gy D30% <17.4Gy	Rectum	SBRT: D1cc <23.4Gy HDR BT: V100% prescribed dose 0 cc D1cc <20.2Gy D2cc <17.4Gy	Bowel	SBRT: D1cc <30Gy D5cc <18.1Gy	Feasibility of randomisation	Acute and late clinician and PROM assessed toxicity
Hruby (NCT03073278)	SBRT	36Gy/6# 38Gy/6#	Bladder	D0.1cc ≤33Gy D0.5cc ≤28 Gy D1cc ≤24Gy D2cc ≤ 18Gy	Urethra_PRV (2 mm)	Urethra Dmax <33Gy Urethra PRV Dmax <36Gy	Rectum	D0.1cc ≤33Gy D0.5cc ≤28 Gy D1cc ≤24Gy D2cc ≤ 18Gy			Feasibility and safety of SBRT dose escalation, assessed by dose limiting toxicity (grade 3+ GU/GI acute and late (1 year) toxicity)	Toxicity
Kang (ACTRN12625000224426)	SBRT	25Gy in 5# + SIB 35Gy	Bladder	Dmax ≤36.75Gy V35Gy ≤5% V32Gy ≤10% V28Gy ≤15% V18.1Gy ≤40%	Urethra_PRV (1 mm)	Dmax ≤34Gy	Rectum	Dmax ≤30Gy V28Gy ≤12% V25Gy ≤5 cc V18.1Gy ≤40%	Bowel	Dmax ≤30Gy V25Gy ≤5 cc	Acute GU/GI toxicity and acute QoL	Late GU/GI toxicity bPFS ADT free survival Late QoL
Majewski (NCT06201078)	SBRT	33.75Gy/5#	Bladder	Dmax ≤35.4Gy (≤34.8Gy optimally) D30% <15Gy	Urethra	Dmax ≤35.4Gy (≤34.8Gy optimally) D30% <15Gy	Rectum	Dmax ≤34.8Gy (≤33.8Gy optimally) D30% <15Gy			Acute and late (2 year) G3+ GU/GI toxicity	Acute/late G2+ GU/GI toxicity bPFS Local control RFS MFS OS QoL
Pasquier (NCT03438552)	SBRT	36Gy/6#	Bladder_Wall	V27Gy <5 cc V12 < 15%	Urethra_PRV (2 mm)	Dmax <39Gy V24Gy <30% V36Gy <1 cc	Rectal_Wall	V27Gy <2 cc V12Gy <20%			Biochemical RFS	Acute and late GU/GI toxicity PFS OS QoL
Taggar (NCT05597852)	SBRT	25Gy in 5# + SIB 35Gy	Bladder	Dmax ≤36.75Gy V35Gy ≤5% V32Gy ≤10% V28Gy ≤15% V18.1Gy ≤40%			Rectum	Dmax ≤30Gy V28Gy ≤12% V25Gy ≤5 cc V18.1Gy ≤40%	Bowel	Dmax ≤30Gy V25Gy ≤5 cc	Feasibility of spacer placement	Acute/late GU/GI toxicity bPFS ADT free survival QoL
												
Burchardt (NCT05715502)	LDR or HDR BT	LDR BT: 145Gy/1# HDR BT: 26Gy/2#			Urethra	LDR: D10% < 140% D30% <120% HDR: D10% < 125% D30% <115% V150% = 0%	Rectum	LDR: D2cc < 90% D0.1cc < 130% HDR: V100% = 0% D2cc < 80%			PROM assessed GU and ED toxicity and QoL at 24 months	Acute and late GU/GI toxicity
Crook (NCT03246802)	HDR BT	HDR BT in 2#									Late G3+ GU/GI toxicity between 3 and 60 months	Clinican and PROM assessed acute and late toxicity QoL bDFS
Dimnik (NCT06202248)	DaRT seeds										Feasibility and safety	
DiNome (NCT02899221)	HDR BT + hyperthermia										Acute G3+ toxicity	Late toxicity bPFS
Solanki (NCT03312972)	HDR BT	Up to 30Gy in 1–2#	Bladder	1#: D0.1cc <17.8Gy D1cc <14.1Gy 2#: D0.1cc <25.6Gy D1cc <20.2Gy	Urethra	1#: Dmax <22.5Gy D1cc <20.6Gy 2#: Dmax <32.4Gy D1cc <29.7Gy	Rectum	1#: D1cc <14.1Gy 2#: D1cc <20.2Gy			Acute G3+ GU/GI toxicity (phase 1)	Clinician and PROM assessed acute and late toxicity Patterns of failure bPFS PCSS OS QoL (phase 2)

ADT, androgen deprivation therapy; bPFS, biochemical progression-free survival; BT, brachytherapy; Dx% or cc, dose to x% or cc [of the volume]; Dmax, maximum dose [to the volume]; GI, gastrointestinal; GU, genitourinary; HDR BT, high dose rate brachytherapy; IMRT, intensity-modulated radiotherapy; LDR BT, low dose rate brachytherapy; OS, overall survival; PCSS, prostate cancer specific survival; PROM, participant-reported outcome measure; PRV, planning risk volume; QoL, quality of life; reRT, reirradiation; RFS, relapse-free survival; SBRT, stereotactic body radiotherapy; SIB, simultaneous integrated boost; Vx% or Gy, volume receiving x% [of the prescribed dose] or dose in Gy.

##### 3.2.3.1 Bladder

There is variation in use of Bladder or Bladder_Wall structures. Near-maximum Bladder/Bladder_Wall constraints at or within ≤105% of prescription dose in 5 fractions are used in 3 SBRT studies. Five studies use a range of volumetric constraints for Bladder/Bladder_Wall. The F-SHARP HDR brachytherapy study uses a near-maximum constraint of 17.8Gy in 1 fraction or 25.6Gy in 2 fractions to Bladder.

##### 3.2.3.2 Urethra

There is variation in use of Urethra or Urethra_PRV structures. Two SBRT studies use near-maximum Urethra_PRV constraints of 34-39Gy in 5–6 fractions. The F-SHARP brachytherapy study uses a near-maximum constraint of 22.5Gy in 1 fraction or 32.4Gy in 2 fractions to Urethra. The RO-PIP study uses a volumetric constraint for both SBRT (5 fractions) and brachytherapy (2 fractions) of 24.2Gy to 10% of Urethra.

##### 3.2.3.3 Rectum

Five SBRT studies use a range of volumetric constraints for Rectum/Rectal_Wall with/without near-maximum constraints. For brachytherapy, RO-PIP and F-SHARP use a 2-fraction constraint of 20.2Gy to 1 cc Rectum and Solanki uses a 1-fraction constraint of 14.1Gy to 1 cc Rectum.

##### 3.2.3.4 Bowel

The RO-PIP, Kang and Tagger studies include Bowel constraints for SBRT of 30Gy in 5 fractions to 1 cc Bowel and 18.1–25Gy to 5 cc Bowel.

#### 3.2.4 Endpoints

The majority of studies have toxicity related primary endpoints (typically ≥grade 3 acute/late GU/GI toxicity), except the GETUG AFU 31 SBRT study by Pasquier et al., which has an RFS endpoint, the RO-PIP study, which has feasibility of randomisation between SBRT and brachytherapy and the Taggar study, which has feasibility of rectal spacer insertion. Secondary endpoints contain disease and toxicity endpoints and most studies also utilise participant-reported outcome measures (PROMs) and evaluate QoL.

## 4 Discussion

This literature review summarises the available evidence from published and ongoing prospective studies relating to treatment planning approaches and toxicity outcomes for patients with locally recurrent prostate cancer treated with SBRT and brachytherapy reirradiation. Given the predominance of retrospective data in this field, focusing on prospective studies provides a higher level of evidence regarding delineation, treatment planning, dosimetry and toxicity assessment.

The optimum salvage therapy for locally recurrent prostate cancer is unknown but disease related outcomes appear similar between prostatectomy, cryotherapy, HIFU, brachytherapy and SBRT, with 5-year relapse-free survival estimated to range from 50 to 60% in the MASTER meta-analysis [5]. However, the MASTER meta-analysis suggested that reirradiation with brachytherapy or SBRT may offer a lower risk of severe toxicities, especially genitourinary (GU) toxicity (4.2–8% vs 15–23%), rates which are in line with the clinical trial and prospective study data evaluated here. Identifying the optimum salvage therapy through a randomised comparison is likely to be challenging, given potential lack of equipoise driven by patient and clinician preference for a particular treatment and the need for patients to be suitable for a general anaesthetic and invasive procedure. The ongoing RO-PIP study recently reported its primary endpoint and was unable to demonstrate feasibility of recruitment to a randomised study of SBRT vs HDR brachytherapy [40], [41]. Future studies focused on evaluating efficacy and toxicity impacts from salvage therapies should therefore consider incorporation of patient, clinician and/or treating centre choice of treatment.

The optimum target volume for prostate reirradiation remains uncertain, and both focal and whole gland approaches with/without SIB to focal lesions were utilised across SBRT and brachytherapy studies. Conflicting views were observed in previous Delphi studies, with either focal or whole gland considered options for brachytherapy and focal treatment preferred for SBRT [9], [10]. Greater availability of multiparametric MRI and PET-CT can help identify localised recurrences, thereby facilitating focal or SIB within a whole gland approach. In the FORECAST study, MRI targeted biopsy of visible lesions diagnosed the majority of recurrent cancer but it was noted that 3 out of 5 participants had additional tumour in other quadrants and systematic biopsy before considering focal salvage treatment is recommended [42]. It is possible that focal treatments may reduce risk of toxicity through a smaller target volume and, in general, ≥grade 2 GU toxicity appeared to be lower in focal compared with whole gland SBRT/brachytherapy studies. Two whole gland LDR brachytherapy studies were associated with relatively high rates of grade 3 and even grade 4 toxicity events (1 study assessed toxicity using CTCAE and 1 used RTOG), although these could also have been influenced by the delivered dose, similar to that utilised for primary LDR brachytherapy, and lack of OAR constraints [20], [27]. Most studies utilised CTCAE for toxicity assessment but some used RTOG, which could result in differences in interpretation of toxicity severity [43]. Delivery of a lower dose to the whole gland plus SIB to a focal lesion could optimally facilitate treatment of non-visible disease while minimising risks of toxicity [13]. In addition to target volume, toxicity is also likely to be influenced by tumour location, especially proximity to urethra or rectum, delivered dose, OAR constraints and use of PRVs. Regardless of the target volume used, dose escalation should be approached with caution given that in the Patel phase I study, focal SBRT using 42.5Gy in 5 fractions resulted in grade 3 GU toxicity for both participants [30]. In addition, some grade 2–3 late GI toxicity events were seen in patients with rectal spacers [17], [30]. While a causal relationship cannot be inferred from these data between GI complications and spacer placement within already fibrotic tissues, caution regarding use of spacers is suggested for prostate reirradiation. There was variation in approaches to such aspects of patient preparation for SBRT studies, making it difficult to interpret impact these had on toxicity. Most studies utilised empty rectum and full bladder, which is a similar approach to that taken for prostate SBRT in the primary setting.

The most appropriate dose-fractionation schedules also remain to be determined, with no clear consensus in previous Delphi studies for SBRT and brachytherapy [9], [10]. Multicentre retrospective data suggest that better biochemical control may be obtained from SBRT schedules which deliver a biologically effective dose >120Gy (corresponding to >30Gy in 5 fractions with an α/β of 2Gy) [44]. In the primary disease setting, data from external beam radiotherapy and brachytherapy boost studies suggest that a dose-response relationship exists, and this may also translate to the recurrence setting [45], [46], [47]. Most published and ongoing SBRT studies evaluated here used 30–40Gy in 5–6 fractions delivered on alternate days, which perhaps represents a reasonable balance between efficacy and toxicity especially where higher doses are delivered as focal or SIB treatments. For primary prostate SBRT, 40Gy to CTV and 36.25Gy to PTV in 5 fractions was used in the PACE-B study, an approach utilised for focal treatments in the salvage setting in the RO-PIP study [40], [48]. Regarding salvage brachytherapy, both single and multifraction HDR brachytherapy schedules were used. In the primary setting, 2-fraction HDR brachytherapy appears to be more effective than single-fraction treatment, and ongoing studies, such as F-SHARP and RO-PIP, may provide insights into whether this relationship exists in the salvage setting [39], [40], [49].

No consensus regarding OAR constraints for reirradiation SBRT was reached in a previous Delphi study nor whether these should be adjusted depending on previously delivered dose [10]. In a brachytherapy reirradiation Delphi study, consensus was reached that OAR constraints should not depend on previous doses although no specific constraints were considered [9]. Most brachytherapy studies did not include Bladder/Bladder_Wall constraints, which perhaps reflects the rapid dose fall-off which is expected and is consistent with recommendations for primary brachytherapy [50]. A previous literature review made recommendations for both near-maximum and volumetric OAR constraints for reirradiation SBRT and brachytherapy, based on prospective studies included in this review [13]. However, the most appropriate OAR constraints for prostate reirradiation are unknown and could not be met for all participants in several studies evaluated here where planned doses were reported. There was considerable variation across SBRT and brachytherapy studies regarding approaches to OAR constraints. Several studies utilised near-maximum small volume constraints close to or at prescription doses, an approach often utilised in reirradiation [51]. Other studies used larger volume constraints, which may be relevant given the parallel nature of OARs such as rectum and bladder. No published nor ongoing study (except RO-PIP) utilised cumulative constraints in EQ.D2 accounting for previously delivered dose, instead describing physical doses which applied to the reirradiation treatment alone. In addition to OAR constraints, prospective studies should therefore include information about previously delivered, planned and cumulative doses (in EQ.D2) and toxicities, in line with recent recommendations regarding standardised reporting for reirradiation [52], [53]. Such studies provide valuable opportunities to prospectively validate OAR constraints and better understand relationships between the volume, and spatial distribution, of dose and toxicity. In addition to dosimetry, patient-level factors such as baseline symptoms are also likely to influence toxicity, as highlighted in the PACE-B study [54]. Based on the studies evaluated here, reirradiation should be used with caution or avoided in patients with significant pre-existing GU/GI toxicity, especially ≥grade 3 toxicity, and consideration should be given to stipulating minimum urinary function.

This review has several limitations. Heterogeneity in target volumes, OAR definitions, dose-fractionation schedules and reporting of OAR constraints and planned doses and variation in follow up duration and differences in toxicity assessment made comparisons between studies challenging. Limited data is available for ongoing studies, especially for brachytherapy studies. This review was not conducted according to Preferred Reporting Items for Systematic Reviews and Meta-analyses (PRISMA) guidelines. However, we performed a comprehensive and updated literature search using PubMed and ClinicalTrials.gov to identify all relevant prospective studies and clinical trials addressing prostate reirradiation planning.

## 5 Conclusion

Prostate reirradiation using SBRT or brachytherapy can be used for treatment of locally recurrent prostate cancer, with acceptable GU and GI toxicity rates observed in the majority of prospective studies. There remains uncertainty regarding optimum dose-fractionation schedules, target volumes and OAR constraints and how these influence toxicity. Treatment is recommended within prospective studies or registries with robust target definition, treatment delivery and comprehensive assessment of toxicity so that evidence to optimise treatment can be generated.

## CRediT authorship contribution statement

**Finbar Slevin:** Writing – review & editing, Writing – original draft, Methodology, Formal analysis, Data curation, Conceptualization. **Brad Warkentin:** Writing – review & editing, Data curation. **Jeremy Hansen:** Writing – review & editing. **Ann M. Henry:** Writing – review & editing. **Louise J. Murray:** Writing – review & editing. **Donna H. Murrell:** Writing – review & editing. **Wee Loon Ong:** Writing – review & editing, Data curation. **David Pasquier:** Writing – review & editing, Data curation, Conceptualization.

## Funding

This work was supported by Cancer Research UK (RRCOER-Jun24/100004).

## Declaration of competing interest

Finbar Slevin reports a relationship with National Institute for Health and Care Research that includes: funding grants. Finbar Slevin reports a relationship with National Institute for Health and Care Research that includes: funding grants. Finbar Slevin reports a relationship with Cancer Research UK that includes: funding grants. Finbar Slevin reports a relationship with Royal College of Radiologists that includes: funding grants. Jeremy Hansen reports a relationship with Siemens Healthineers that includes: employment. Ann Henry reports a relationship with Cancer Research UK that includes: funding grants. Ann Henry reports a relationship with European Association of Urology that includes: board membership. Louise Murray reports a relationship with Cancer Research UK that includes: funding grants. Louise Murray reports a relationship with National Institute for Health and Care Research that includes: funding grants. Louise Murray reports a relationship with The Jon Moulton Charity Trust that includes: funding grants.
